# Behavioral Relaxation Training within an ABA Framework: A Multiple Baseline Study on Adults with Anxiety Disorders

**DOI:** 10.1192/j.eurpsy.2026.10244

**Published:** 2026-07-31

**Authors:** V. Pascale, N. Varrucciu, R. Di Sarro

**Affiliations:** 1 NDD, abafordisability, Salerno; 2 Mental Health, AUSL Bologna, Bologna, Italy

## Abstract

**Introduction:**

Anxiety disorders represent one of the most prevalent mental health conditions in adulthood, often associated with significant impairment in daily functioning and well-being. Standard treatments include pharmacological and cognitive-behavioral approaches, but additional behavioral interventions may enhance outcomes. Behavioral Relaxation Training (BRT), grounded in Applied Behavior Analysis (ABA), focuses on the systematic acquisition of relaxation postures and breathing patterns, offering a direct behavioral strategy to reduce physiological arousal and anxiety symptoms.

**Objectives:**

This study aimed to investigate the efficacy of BRT in reducing anxiety symptoms and improving relaxation behaviors in adults diagnosed with anxiety disorders, using a controlled experimental design.

**Methods:**

A multiple baseline design across three caregivers was employed to demonstrate experimental control. Each participant engaged in structured BRT sessions designed to teach and generalize relaxation postures and regulated breathing. Outcome measures included the Behavioral Relaxation Scale (BRS) as a direct behavioral index of relaxation, alongside standardized clinical instruments such as the Hamilton Anxiety Rating Scale (HAM-A) and the Clinician-Administered PTSD Scale (CAPS), following procedures described in previous research (Lundervold et al., 2013).

**Results:**

Findings revealed consistent reductions in anxiety severity and significant improvements in relaxation behavior across all participants. Decreases in HAM-A and CAPS scores paralleled improvements in BRS performance, indicating both subjective and behavioral gains. Treatment effects were maintained at follow-up, supporting the stability of outcomes over time.

**Conclusions:**

Preliminary evidence suggests that BRT, when embedded within an ABA framework, represents an effective behavioral intervention for adults with anxiety disorders. The approach promotes emotional regulation, reduces physiological tension, and complements existing therapeutic models. Further studies with larger samples are warranted to strengthen the empirical base and explore generalization across broader clinical populations

**Disclosure of Interest:**

None Declared

